# Complete chloroplast genome sequences of the medicinal plant *Piper hancei*

**DOI:** 10.1080/23802359.2021.1967217

**Published:** 2021-08-27

**Authors:** Lvshui Zhang, Xiaokang Hu, Mu Liu

**Affiliations:** a College of Landscape Architecture and Arts, Jiangxi Agricultural University, Nanchang, China; b Gannan Arboretum, Ganzhou, China

**Keywords:** Chloroplast genome, *Piper hancei*, molecular markers, SSR, phylogeny

## Abstract

Chloroplast genome sequences have been used in phylogenetic and population genetics studies. Here, we assembled the chloroplast genome of *Piper hancei* Maxim. that is a traditional Chinese medicine. The genome length was 161,476 bp and included a pair of inverted repeats of 27,058 bp, a large single-copy region of 89,144 bp and a small single-copy region of 18,216 bp. It contained 113 different genes, including 79 protein-coding genes, 30 transfer RNA (tRNA), and four ribosomal RNA genes. Moreover, we also identified 82 SSRs. The phylogenetic inference based on the whole chloroplast genome of 20 taxa showed *P. hancei* was sister to *P. kadsura*.

*Piper hancei* Maxim. is used as a traditional Chinese medicine within the family Piperaceae and is natively distributed in Fujian, Guangdong, Guangxi, Guizhou, Hunan, Yunnan, and Zhejiang provinces (Li and Hah). It is mainly used as a medicine to relieve pain, dispel wind and swelling, and treat rheumatic arthritis, weakness of the back and knees, and cough and asthma (Li and Hah). *Piper hancei* is also used as gardening plant in tropical and subtropical regions of China. At present, there was less genetic information about this species, which brings difficulties to cultivation and utilization. In this study, we sequenced and analyzed the chloroplast genome of *P. hancei* based on the next-generation sequencing method (Dong, Sun, et al. 2021; Dong, Xu, et al. 2021; Wang et al. 2021). The objective of this study was to describe the chloroplast genome structure and feature for this species.

Fresh and young leaves of *P. hancei* was collected from Gannan Arboretum, Jiangxi, China (25°51′10″∼114°22′25″), which was introduced from Guangdong. A specimen and DNA were deposited at the herbarium of Jiangxi Agricultural University under the voucher number of LM750014. Total genomic DNA was isolated using a mCTAB protocol (Li et al. 2013) for constructing a 350 bp insert library and sequencing on an Illumina Hiseq X ten platform. Raw data were qualified using Trimmomatic (Bolger et al. 2014) and the chloroplast genome was assembled with GetOrganelle (Jin et al. 2020) using clean data. The complete chloroplast genome was annotated with Plann using *P. auritum* (GenBank accession number: KY085906) as reference (Huang and Cronk 2015). The annotated chloroplast genome of *P. hancei* has been deposited into GenBank with the accession number of MZ046380.

The chloroplast genome of *P. hancei* was a circular DNA molecule of 161,476 bp and had a typical quadripartite structure with a pair of inverted repeats of 27,058 bp, a large single-copy region of 89,144 bp and a small single-copy region of 18,216 bp. The GC content was 38.3%, which was similar to other Piperaceae species (Cai et al. 2006). The *P. hancei* chloroplast genome encoded 113 different genes, including 79 protein-coding genes, 30 transfer RNA (tRNA), and 4 ribosomal RNA genes. Eighteen genes contain introns, 16 of which contain one intron and two (*clpP* and *ycf3*) with two introns.

Simple sequence repeats in the *P. hancei* chloroplast genomes were detected using GMAT (Wang and Wang 2016) with the minimum repeats of mono-, di-, tri-, tetra-, penta- and hexa-nucleotides being set to 10, 5, 4, 3, 3, and 3, respectively. A total of 82 perfect chloroplast genome SSR were identified. The number of mono-, di-, tri-, tetra-, penta- and hexa-nucleotides were 49, 13, 8, 7, 4 and one, respectively.

To estimate the phylogenetic relationships of *P. hancei* with other *Piper* species. Phylogenetic analysis was performed using the whole chloroplast genome sequence from 20 Piperales species. Whole chloroplast genome sequences were aligned with MAFFT v7 (Katoh and Standley 2013) and ambiguous alignment regions were trimmed by trimAl v1.2 (Capella-Gutiérrez et al. 2009). Maximum Likelihood (ML) tree was preformed using RAxML-NG (Kozlov et al. 2019). The GTR + G was chosen as the best-fit DNA substitution model according to the Akaike Information Criterion correction in ModelFinder (Kalyaanamoorthy et al. 2017). The reconstructed phylogeny revealed that *P. hancei* was sister to *P. kadsura* (Figure 1).

**Figure 1.**
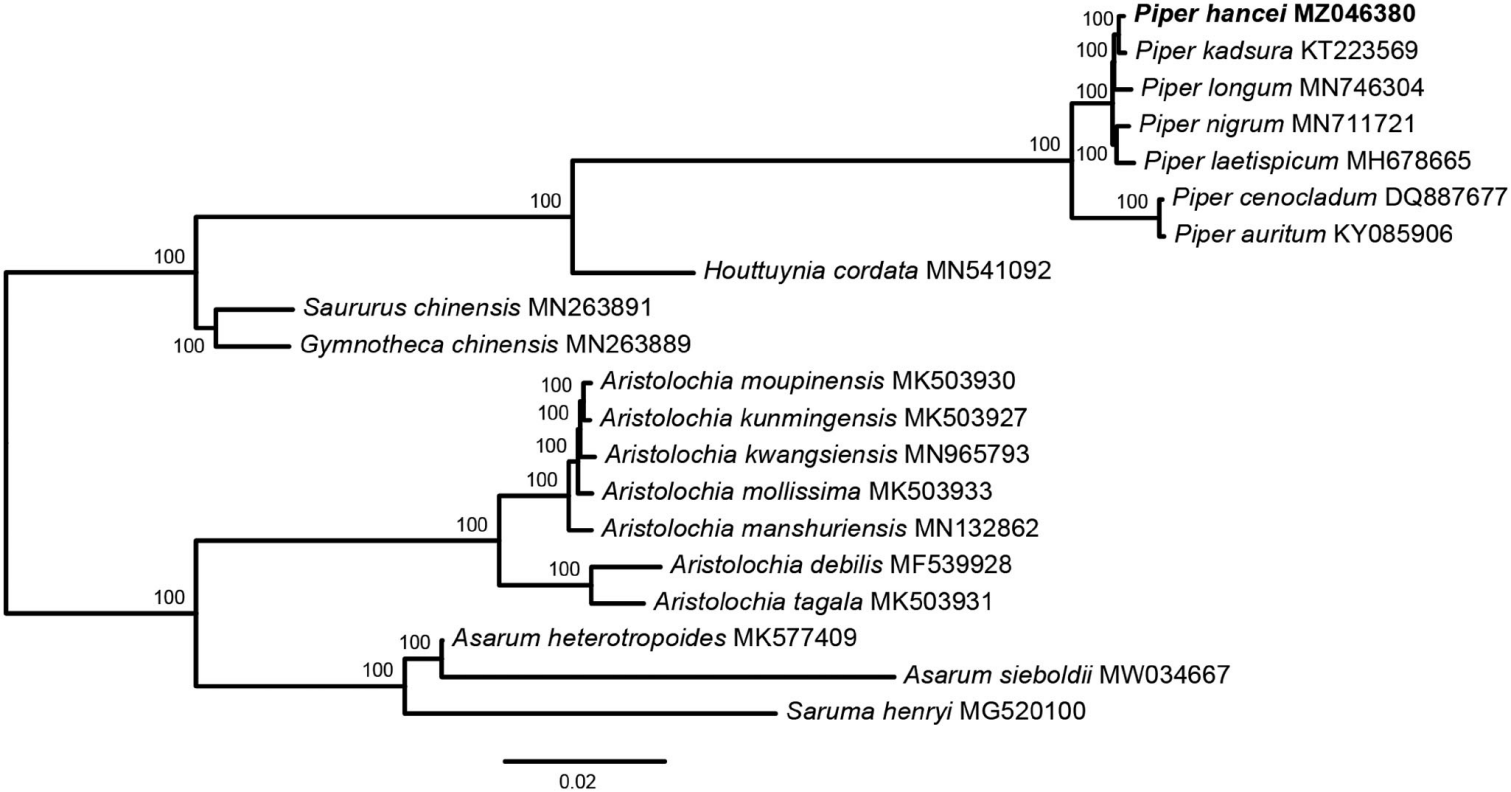
Maximum likelihood tree of Piperales based on the complete chloroplast genome sequences. ML bootstrap support value presented at each node.

## Data Availability

The genome sequence data that support the findings of this study are openly available in GenBank of NCBI at (https://www.ncbi.nlm.nih.gov/) under the accession no. MZ046380. The associated BioProject, SRA, and Bio-Sample numbers are PRJNA725313, SRR14328341, and SAMN18875907, respectively.

## References

[CIT0001] Bolger AM, Lohse M, Usadel B. 2014. Trimmomatic: a flexible trimmer for Illumina sequence data. Bioinformatics. 30(15):2114–2120. 24695404 10.1093/bioinformatics/btu170 PMC4103590

[CIT0002] Cai Z, Penaflor C, Kuehl JV, Leebens-Mack J, Carlson JE, dePamphilis CW, Boore JL, Jansen RK. 2006. Complete plastid genome sequences of *Drimys*, *Liriodendron*, and *Piper*: implications for the phylogenetic relationships of magnoliids. BMC Evol Biol. 6:77. 17020608 10.1186/1471-2148-6-77 PMC1626487

[CIT0003] Capella-Gutiérrez S, Silla-Martínez JM, Gabaldón T. 2009. trimAl: a tool for automated alignment trimming in large-scale phylogenetic analyses. Bioinformatics. 25(15):1972–1973. 19505945 10.1093/bioinformatics/btp348 PMC2712344

[CIT0004] Dong W, Sun J, Liu Y, Xu C, Wang Y, Suo Z, Zhou S, Zhang Z, Wen J. 2021. Phylogenomic relationships and species identification of the olive genus Olea (Oleaceae). J System Evol.doi: 10.1111/jse.12802

[CIT0005] Dong W, Xu C, Liu Y, Shi J, Li W, Suo Z. 2021. Chloroplast phylogenomics and divergence times of Lagerstroemia (Lythraceae). BMC Genom. 22(1):434. 10.1186/s12864-021-07769-x PMC8191006 34107868

[CIT0006] Huang DI, Cronk QCB. 2015. Plann: A command-line application for annotating plastome sequences. Appl Plant Sci. 3(8):1500026. 10.3732/apps.1500026 PMC4542940 26312193

[CIT99717579] Jin J-J, Yu W-B, Yang J-B, Song Y, dePamphilis CW, Yi T-S, Li D-Z. 2020. GetOrganelle: a fast and versatile toolkit for accurate de novo assembly of organelle genomes. Genome Biol. 21(1):241.doi:10.1186/s13059-020-02154-5. PMC: 32912315 32912315 PMC7488116

[CIT0008] Kalyaanamoorthy S, Minh BQ, Wong TKF, von Haeseler A, Jermiin LS. 2017. ModelFinder: fast model selection for accurate phylogenetic estimates. Nat Methods. 14(6):587–589. 28481363 10.1038/nmeth.4285 PMC5453245

[CIT0009] Katoh K, Standley DM. 2013. MAFFT multiple sequence alignment software version 7: improvements in performance and usability. Mol Biol Evol. 30(4):772–780. 23329690 10.1093/molbev/mst010 PMC3603318

[CIT0010] Kozlov AM, Darriba D, Flouri T, Morel B, Stamatakis A. 2019. RAxML-NG: a fast, scalable and user-friendly tool for maximum likelihood phylogenetic inference. Bioinformatics. 35(21):4453–4455. 31070718 10.1093/bioinformatics/btz305 PMC6821337

[CIT0011] Li J, Wang S, Jing Y, Wang L, Zhou S. 2013. A modified CTAB protocol for plant DNA extraction. Chinese Bullet Bot. 48:72–78.

[CIT0012] Li S-M, Hah G-Q. 1987. Studies on the chemical constituents of *Piper hancei* Maxim III. J Integr Plant Biol. 29(3):293–296. 3661206

[CIT0013] Wang X, Wang L. 2016. GMATA: an integrated software package for genome-scale SSR mining, marker development and viewing. Front Plant Sci. 7:1350. 27679641 10.3389/fpls.2016.01350 PMC5020087

[CIT0014] Wang M, Wang X, Sun J, Wang Y, Ge Y, Dong W, Yuan Q, Huang L. 2021. Phylogenomic and evolutionary dynamics of inverted repeats across *Angelica* plastomes. BMC Plant Biol. 21(1):26. 33413122 10.1186/s12870-020-02801-w PMC7792290

